# Pericapsular nerve group block for total hip arthroplasty: A systematic review

**DOI:** 10.1177/03000605261476867

**Published:** 2026-08-25

**Authors:** Daniela Bravo, Hernán Arancibia, Maibelyn Manzanilla, Álvaro Jara, Samita Pirotesak, Julián Aliste, De Q Tran

**Affiliations:** 1 Department of Anesthesiology and Perioperative Medicine, 14655 University of Chile, Hospital Clínico Universidad de Chile, Chile; 2 Department of Anatomy and Legal Medicine, Faculty of Medicine, 14655 University of Chile, Chile; 3 Department of Anesthesiology, 65106 McGill University, Montreal General Hospital, Canada; 4 Department of Anesthesiology, Faculty of Medicine Siriraj Hospital, Mahidol University, Thailand

**Keywords:** Pericapsular nerve group block, hip arthroplasty, nerve block, infiltration anesthesia, acute postoperative pain, early mobilization

## Abstract

**Objective:**

Pericapsular nerve group block spares the main trunk of the femoral nerve, and its use has rapidly expanded from hip fracture surgery to total hip arthroplasty, where preservation of quadriceps function is paramount for early rehabilitation and ambulation. This systematic review investigated the analgesic and motor-sparing benefits of pericapsular nerve group block for total hip arthroplasty.

**Methods:**

This systematic review included randomized trials evaluating pericapsular nerve group block in adult patients undergoing total hip arthroplasty. Databases, including MEDLINE, EMBASE, CINAHL, and Google Scholar, were searched (inception through May 2026). Only randomized trials published in English, with prospective registration and without discrepancies between the registered and reported protocols, were retained for analysis.

**Results:**

Across 10 randomized trials, from an analgesic standpoint, pericapsular nerve group block was found to be noninferior to intrathecal morphine (100 µg) and superior to infrainguinal fascia iliaca block. Pericapsular nerve group block provided similar analgesia and breakthrough opioid consumption compared with suprainguinal fascia iliaca block, anterior quadratus lumborum block, and L4 erector spinae plane block; however, it may improve motor strength during the initial postoperative period. Combined lateral quadratus lumborum block–periarticular local anesthetic infiltration and combined pericapsular nerve group block–periarticular local anesthetic infiltration–lateral femoral cutaneous nerve block provided similar pain at rest and functional outcomes, although lateral quadratus lumborum block–periarticular local anesthetic infiltration achieved lower pain with movement and reduced opioid requirements. Compared with pericapsular nerve group block, periarticular local anesthetic infiltration resulted in lower dynamic pain (first 6 h) and static pain (first 48 h), and adding pericapsular nerve group block to a robust periarticular local anesthetic infiltration regimen provided minimal incremental benefit.

**Conclusion:**

Pericapsular nerve group block provided an equianalgesic, motor-sparing alternative to suprainguinal fascia iliaca block, anterior quadratus lumborum block, and lumbar erector spinae plane block for total hip arthroplasty. However, intrathecal morphine may provide comparable analgesia to pericapsular nerve group block. Furthermore, a robust periarticular local anesthetic infiltration regimen may outperform pericapsular nerve group block. Consequently, future large-scale trials are required to elucidate the benefits of pericapsular nerve group block compared with intrathecal morphine (for inpatient total hip arthroplasty) and periarticular local anesthetic infiltration (for inpatient and outpatient total hip arthroplasty).

**INPLASY registration number:**

INPLASY202670024.

## Introduction

Described in 2018, pericapsular nerve group (PENG) block targets the articular branches of the femoral and accessory obturator nerves along the pubic ramus, between the anterior inferior iliac spine and the iliopubic eminence, dorsal to the iliopsoas muscle and its tendon.^1^ Because PENG block spares (in theory) the main trunk of the femoral nerve, its use has rapidly expanded from hip fracture surgery to total hip arthroplasty (THA), where preservation of quadriceps function is paramount for early rehabilitation and ambulation.^2^ Since 2018, multiple randomized trials have investigated the analgesic benefits of PENG block for THA. Unfortunately, most of these trials have demonstrated irregularities (e.g. retrospective trial registration) or potential investigator bias (e.g. changes in the primary outcome/sample size after the start of patient recruitment). These methodological shortcomings inevitably result in a prohibitive knowledge gap.

Consequently, in order to summarize the best available evidence regarding the benefits of PENG block for THA, this systematic review focuses exclusively on randomized trials published in English that were prospectively registered and displayed no discrepancies between the registered and reported study protocols.

## Methods

This systematic review was conducted and reported in accordance with the Preferred Reporting Items for Systematic Reviews and Meta-Analyses (PRISMA) 2020 statement and its explanation and elaboration document.^3,4^ The completed PRISMA 2020 checklist is provided as Supplementary Material (uploaded as Research Data). This study was registered after study completion with INPLASY (https://inplasy.com/; registration number: INPLASY202670024; doi:10.37766/inplasy2026.7.0024). The literature search for this review was conducted by six coauthors (DB, HA, MM, AJ, SP, and JA) across the MEDLINE, EMBASE, CINAHL, and Google Scholar databases (from inception through the first week of May 2026). The terms “pericapsular nerve group block” and “hip arthroplasty” were searched to identify English-language randomized trials comparing PENG block with sham/no block/other blocks. Alternative terms such as “PENG block” and “hip surgery” were also queried for completeness. The reference lists of all trials identified in the initial search were subsequently examined to identify additional randomized trials.

We excluded trials investigating PENG block in surgical settings other than THA (i.e. hip fracture repair and hip arthroscopy). Furthermore, only trials comparing PENG block with a control, alternative blocks, or intrathecal morphine were retained for analysis (trials in which all study groups and all patients received PENG block were excluded). Moreover, in order to minimize investigator bias, we elected to exclude all randomized trials lacking proper prospective trial registration, blinded assessment, or displaying discrepancies between the registered and published protocols. Any disagreement among the six coauthors was resolved by the senior author (DQT).

For each retained trial, validity was further assessed using the Cochrane Risk of Bias Tool. Six domains were evaluated by two coauthors (DB and HA): adequacy of sequence generation, allocation concealment, blinding, handling of incomplete outcome data, selective outcome reporting, and other sources of bias (e.g. study design issues, early trial termination, and baseline imbalance between study groups). Thereafter, each domain was categorized as “yes” (green; low risk of bias), “no” (red; high risk of bias), or “unclear” (yellow; unclear risk of bias). Because rigorous criteria for trial retention were already applied, no additional exclusions were planned in the event of a high risk of bias in any of the six domains. Any disagreement between the two coauthors was resolved by the senior author (DQT).

Subsequently, six coauthors (DB, HA, MM, AJ, SP, and JA) independently extracted the clinical data pertaining to static/dynamic pain, breakthrough opioid consumption, quadriceps paralysis, and adverse effects from each eligible study. Any disagreement was resolved by the senior author (DQT).

## Results

**Figure 1.**
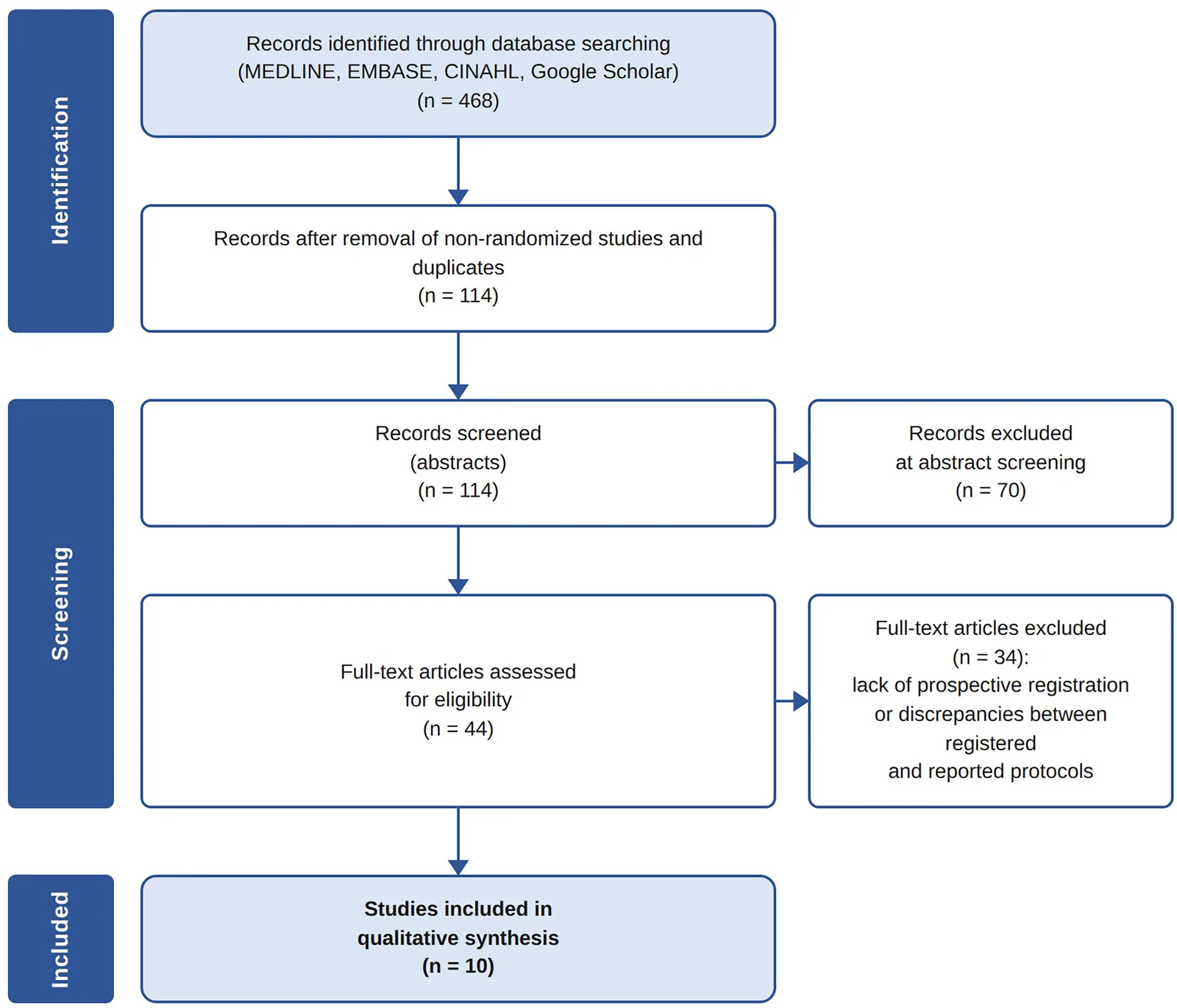
PRISMA 2020 flow diagram of study identification, screening, and inclusion.

**Figure 2.**
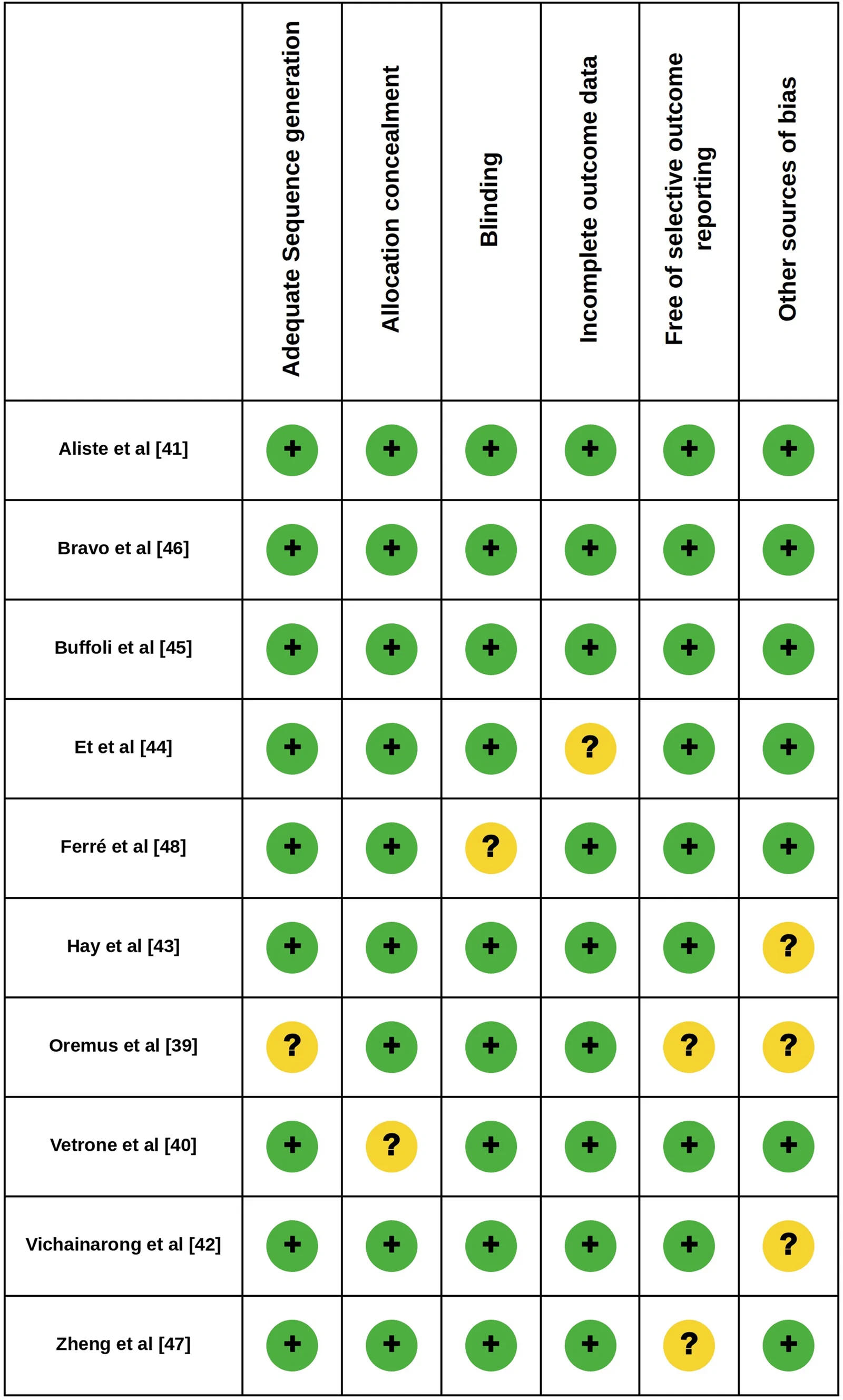
Risk of bias summary of randomized trials investigating pericapsular nerve group block for total hip arthroplasty.

Our search strategy identified 468 studies across the different databases. Removal of nonrandomized trials and duplicates left 114 potentially eligible studies. After screening the abstracts, we excluded 70 studies that did not meet the inclusion criteria. The remaining 44 trials underwent full-text review for eligibility. The majority (34) of randomized trials lacked proper registration or displayed serious discrepancies between the registered and reported protocols. For the sake of completeness, these excluded studies (and the reasons for their exclusion) are presented in Table 1.^5–38^ A total of 10 trials were retained for analysis (Tables 2 and 3).^39–48^ The study selection process is summarized in Figure 1 (PRISMA 2020 flow diagram). The risk of bias summary of the retained trials is presented in Figure 2.

PRISMA: Preferred Reporting Items for Systematic Reviews and Meta-Analyses.

**Table 1.** Discarded studies investigating PENG block for THA.

Trial	Reason for exclusion
Abd El-Gawad et al. (2025)^5^	No trial registration
Adbelrahman et al. (2025)^6^	Retrospective registration
Abdelsalam et al. (2024)^7^	No trial registration
Abdou et al. (2024)^8^	No trial registration
Carella et al. (2023)^9^	Discrepancy between registered sample size, calculated sample size (in manuscript), and number of randomized cases Discrepancy between registered and reported secondary outcomes
Cho et al. (2025)^10^	Discrepancy between registered and reported primary outcome Discrepancy between registered and reported secondary outcomes
Choi et al. (2022)^11^	Discrepancy between registered and reported secondary outcomes
Chung et al. (2022)^12^	Registration incomplete (no reported period of patient recruitment) Discrepancy between registered and reported secondary outcomes
Cui et al. (2024)^13^	Discrepancy between registered and reported secondary outcomes
Domagalska et al. (2023)^14^	Retrospective registration
Duan et al. (2023)^15^	Discrepancy between registered and reported intervention groups and techniques
Elnefiawy et al. (2023)^16^	Discrepancy between registered sample size, calculated sample size (in manuscript), and number of randomized cases Discrepancy between registered and reported secondary outcomes
Gonabal et al. (2023)^17^	Retrospective registration
Hanauer et al. (2025)^18^	Discrepancy between registered sample size, calculated sample size (in manuscript), and number of randomized cases
Hu et al. (2022)^19^	Sham PENG block not included in the registry Discrepancy between registered and reported secondary outcomes
Hu et al. (2025)^20^	Discrepancy between registered and reported secondary outcomes
Iglesias et al. (2022)^21^	No trial registration
Kalashetty et al. (2022)^22^	Retrospective registration
Kamel et al. (2024)^23^	No trial registration
Kukreja et al. (2023)**^2^**^4^	Discrepancy between registered sample size, calculated sample size (in manuscript), and number of randomized cases Changes in secondary outcomes after trial initiation
Kücúk et al. (2024)^25^	Retrospective registration
Lee et al. (2024)^26^	Discrepancy between registered and reported sample sizes Change in primary outcome after trial initiation Discrepancy between registered and reported secondary outcomes
Liang et al. (2023)^27^	Discrepancy between registered and reported sample sizes Discrepancy between registered and reported primary outcome Discrepancy between registered and reported secondary outcomes
Lin et al. (2022)^28^	Discrepancy between registered and reported sample sizes
Medhat et al. (2023)^29^	Retrospective registration
Pascarella et al. (2021)^30^	Status changed from “non-randomized” to “randomized” after the end of patient recruitment.
Svraka et al. (2024)^31^	No trial registration
Turkan. (2024)^32^	Retrospective registration
Vamshi et al. (2023)^33^	Discrepancy between registered and reported secondary outcomes
Wang et al. (2023)^34^	Retrospective registration
Ye et al. (2023)^35^	Retrospective registration
Zheng et al. (2021)^36^	Retrospective registration
Zhu et al. (2024)^37^	Discrepancy between registered and reported patient recruitment periods; according to registry, patient enrolment predates registration Discrepancy between registered and reported secondary outcomes
Erdem et al. (2026)^38^	Change in definition of the primary outcome after the start of patient recruitment Change in sample size after the end of patient recruitment

PENG: pericapsular nerve group; THA: total hip arthroplasty.

**Table 2.** Retained trials investigating PENG block for total hip arthroplasty.

Authors	N	Study groups	Surgical approach	Block timing	Type of anesthesia	Motor block assessment	Primary outcome	Secondary outcomes	Main results	Comments
Aliste et al.^41^	40	PENG block: Levobupivacaine 0.5% + epinephrine 5 ug/ml, 20 mL SIFIB: Levobupivacaine 0.25% + epinephrine 5 ug/ml, 40 mL	Posterolateral	Postoperative	Spinal anesthesia (Isobaric Bupivacaine 10 mg + fentanyl 20μg)	Yes (3, 6, and 24 h) Quadriceps strength (knee extension) 0 = normal (extension against gravity and against resistance) 1 = paresis (extension against gravity but not against resistance) 2 = paralysis (no extension possible) Hip adduction postoperative reduction 0 = normal (0%–20%) 1 = paresis (21%–70%) 2 = paralysis (71%–90%)	Incidence quadriceps motor block at 6 h (paresis or paralysis of knee extension)	Performance time Block-related adverse events Static and dynamic pain scores at 3, 6, 12, 18, 24, 36, and 48 h Morphine consumption (24 and 48 h) Opioid-related side effects Inability to perform physiotherapy (24 and 48 h) due to motor block or pain Length of stay Quadriceps motor block (3 and 24 h) Adduction block (3, 6, and 24 h) Sensory block (3, 6, and 24 h)	PO: PENG block resulted in a lower incidence of quadriceps motor block at 6 h (25% for PENG block and 85% for SIFIB) PENG block showed decreased motor block and sensory block at all measured time points. Dynamic pain scores were lower with SIFIB at 3 and 36 h No differences in terms of block performance time, block-related adverse events, static pain scores, cumulative opioid consumption at 24 and 48 h, opioid-related side effects, ability to perform physiotherapy at 24 and 48 h, and length of stay^a^	
Bravo et al.^46^	60 total	PENG block: Bupivacaine 0.5% + epinephrine 5 ug/ml, 20 mL PAI: Bupivacaine 0.25% + epinephrine 5 ug/mL + ketorolac 30 mg, 60 mL	Posterolateral	Postoperative	Spinal anesthesia (Isobaric Bupivacaine 10 mg + fentanyl 20μg)	Yes (3, 6, and 24 h) 0 = normal (extension against gravity and against resistance) 1 = paresis (extension against gravity but not against resistance) 2 = paralysis (no extension possible) Hip adduction postoperative reduction 0 = normal (0%–20%) 1 = paresis (21%–70%) 2 = paralysis (71%–90%)	Incidence of quadriceps motor block at 3 h (paresis or paralysis of knee extension)	Performance time Block-related adverse events Static and dynamic pain scores at 3, 6, 12, 18, 24, and 48 h Time to first opioid request Morphine consumption (24 and 48 h) Opioid-related side effects Inability to perform physiotherapy (24 and 48 h) due to motor block or pain Length of stay Quadriceps motor block (6 and 24 h) Adduction block (3, 6, and 24 h) Sensory block (3, 6, and 24 h)	PO: no difference between PENG block and PAI (20% vs. 33%, respectively) of quadriceps motor block incidence at 3 h No intergroup differences were found in terms of sensory block or motor block at any of the other time intervals^a^ PAI showed lower static pain scores (all time intervals) and lower dynamic pain scores (3 and 6 h) No differences in terms of block performance time, block-related adverse events, time to first opioid request, cumulative opioid consumption at 24 and 48 h, opioid-related side effects, ability to perform physiotherapy at 24 and 48 h, and length of stay^a^	PAI technique: after insertion of the acetabular component (and before insertion of the femoral stem), 30 mL were infiltrated into deep tissues (i.e. anterior and posterior capsules, gluteus minimus and medius muscles, supraacetabular region, area around the anterior inferior iliac spine, and quadratus femoris muscle all the while avoiding the deep hip external rotator muscle group in order to prevent sciatic nerve block). Before wound closure, the gluteus maximus muscle, iliotibial band, subcutaneous tissues, and skin were infiltrated with the remaining 30 mL
Buffoli et al.^45^	62 total	PENG block: Ropivacaine 0.5% 20 mL + LFCN block: ropivacaine 0.5% 10 mL L4 ESP block: ropivacaine 0.5% 30 mL	Posterolateral	Preoperative (before spinal anesthesia)	Spinal anesthesiaIsobaric Bupivacaine 11mg	Yes (first 24 h) Knee extension or hip adduction or both	Morphine consumption at 24 h	Static and dynamic pain at 6, 12, 24, and 48 h Occurrence of nausea, orthostatic hypotension, chronic pain (at 3 months) Knee extension or hip adduction first 24 h)^a^	PO: no differences in morphine consumption No differences in static or dynamic pain, nausea, orthostatic hypotension, return of bowel function, chronic pain^a^ No differences in motor block^a^	
Et et al.^44^	89 Total	PENG block: 20 mL Bupivacaine 0.5% + sham anterior QLB Anterior QLB: 30 mL Bupivacaine 0.5%, + sham PENG block Intra-articular injection: 60 mL Bupivacaine 0.25% + sham anterior QLB and sham PENG block	Posterolateral	Preoperative (post spinal anesthesia, before surgery)	Spinal anesthesia (Hyperbaric Bupivacaine 0.5%)	Yes (3, 6, 12, and 24 h) Quadriceps strength (knee extension) 0 = normal (extension against gravity and against resistance) 1 = paresis (extension against gravity but not against resistance) 2 = paralysis (no extension possible) Hip adduction postoperative reduction 0 = normal (0%–20%) 1 = paresis (21%–70%) 2 = paralysis (71%–90%)	Maximum severity of static and dynamic pain (NRS) perceived at 3, 6, 8, 12, 24, and 48 h	Time to first opioid Total opioid morphine equivalents consumption in 48 h Muscle strength QoR-40 days 1, 2, and 7 Time to first mobilization Adverse events Satisfaction	PO: PENG block and anterior QLB performed better than intra-articular injection NRS at rest at 3 h and dynamic NRS at 3 and 6 h Anterior QLB and PENG block showed a larger time to first opioid compared to intra-articular injection The analgesic requirement between 0–6 h for PENG block and anterior QLB were lower than intra-articular injection Anterior QLB exhibited less total opioid consumption at 48 in vs. intra-articular injection Decreased quadricep strength with anterior QLB vs. PENG block at 3 h Earlier mobilization with PENG block compared to anterior QLB No differences in hip adduction, QoR-40, satisfaction or adverse events^a^	Surgical drainage was used No sensory block evaluation performed The trial includes fractures and elective THA
Ferré et al.^48^	64 total	PAI: Ropivacaine 0.2%, 80 mL PAI (same) + PENG block: Ropivacaine 0.475%, 20 mL	Posterolateral	Preoperative (before general anesthesia)	General anesthesia	Yes (postoperative day 1) TUG test Adductor force measured via sphygmomanometer	Total morphine consumption at 24 h	Worst postoperative rest pain score at end of PACU stay, between PACU and day 1, between day 1 and day 2 Postoperative opioid consumption was recorded in the PACU, 24 h (day 1) and 48 h (day 2) after surgery Pain induced by the PENGB Motor functional impairment Patient satisfaction Opioid side effects	PO: opioid consumption at 24 h (day 1) were not statically difference between groups No differences in opioid consumption in any measure interval^a^ No differences in worst postoperative pain score at any time point^a^ Pain experienced during the PENG block was low No difference at motor assessment between groups^a^ No differences in patient satisfaction or opioid side effects^a^	PAI technique: distributed equally between the intra-articular and periarticular areas (under the posterior capsule), intermuscular areas (under the rectus femoris, between the gluteus minimus and tensor fasciae lata muscles and under the vastus lateralis muscle) and the subcutaneous area
Hay et al.^43^	106 total	PENG block + LFCN block: Ropivacaine 0.25%, 30 mL (20 mL/10 mL) Lateral QLB: Ropivacaine 0.25%, 30 mL Both PAI: 1 mL/kg of a 100 mL mixture containing: ropivacaine 246.25 mg, epinephrine 0.5 mg, clonidine 80 μg, ketorolac 30 mg; maximal dose 100 mL)	Anterior, posterior and direct lateral	Preoperative	Spinal anesthesia (Bupivacaine 10 mg) General anesthesia if failure of neuraxial technique	Indirectly (time to first ambulation)	Postoperative cumulative opioid consumption at 72 h	Postoperative cumulative opioid consumption at PACU and at 12, 24, 36, 48, and 60 h postoperatively Postoperative pain at rest and dynamic (VAS score) at PACU, 12, 36, 48, 60, and 72 h Time to first ambulation Distance of first ambulation Length of stay Same-day discharge rates Patients recorded outcome measures (HOOS JR and PROMIS-10 Global Health questionnaires) at 1, 2, and 6 weeks Opioid-related side effects	PO: mean opioid consumption differed at 72 h in favor of lateral QLB PENG block consumed more opioids at 36, 48, and 60 h There were no significant differences between treatment arms in average resting and dynamic pain scores, time to ambulation, rate of same-day discharge, length of stay, patient-reported functional outcomes or opioid-related side effects^a^	No PAI description Unbalanced distribution of surgical approaches among groups Possibly underpowered due to loss of follow up after discharge
Oremus et al.^39^	60 total	PENG block: levobupivacaine 0.5% 30 mL + dexamethasone 2 mg Intrathecal morphine: 100 µg	Not specified	Preoperative (after spinal anesthesia)	Spinal anesthesia (isobaric levobupivacaine 0.5% 15 mg)	Yes Straight leg raise at 4, 6, 12 h	3 co-primary outcomes: Maximum pain at rest over 48 h Maximum pain with 60 degrees hip flexion over 48 h Cumulative morphine consumption over 48 h	Straight leg raise at 4, 6, 12 h Incidence of nausea/vomiting and pruritus Minimum systolic and diastolic blood pressure over 24 h	PO: No intergroup differences in maxim pain at rest, maximum pain with hip flexion, cumulative consumption over 48 h No intergroup differences in straight leg raise^a^ No intergroup differences in nausea/vomiting/pruritus/blood pressure^a^	
Vetrone et al.^40^	58 total	PENG block: ropivacaine 0.5% 20 mL + LFCN block: ropivacaine 0.5% 10 mL Infrainguinal fascia iliaca block: ropivacaine 0.5% 20 mL	Direct anterior	Preoperative (after spinal anesthesia)	Spinal anesthesia (hyperbaric bupivacaine 0.5% 0.06 mg/cm height + sufentanil 5 µg)	yes	Degree of residual motor weakness at 6 h (assessed with Medical Research Council Manual Testing Scale)	Static and dynamic pain scores at 6, 24, 48 h Residual motor weakness at 24 and 48 h Hip flexion at 6, 24, and 48 h Opioid consumption Time to first ambulation Side effects	PO: less residual weakness at 6 h with PENG block Less residual weakness at 24 and 48 h; and better hip flexion at 6, 24, 48 h with PENG block PENG block: less static and dynamic pain, less opioid consumption and less nausea/vomiting No intergroup differences in ambulation^a^	
Vichainarong et al.^42^	60 total	PENG block: Bupivacaine 033% + epinephrine 5 μg/mL, 30 mL SIFIB: Bupivacaine 033% + epinephrine 5 μg/mL, 30 mL Both groups received TFPB: Bupivacaine 0.25% + epinephrine 5 μg/mL, 15 mL	Posterior	Preoperative (after general anesthesia)	General anesthesia	Yes TUG test (preoperative and 48 h postoperatively) QMS test, measured in Newton (N) (preoperative and 12 or 24 h postoperatively)	Total fentanyl consumption at 24 h	Intraoperative fentanyl consumption Postoperative fentanyl consumption at PACU, 6 h and 48 h Fentanyl PCA demanded/delivered ratio at 6, 24, and 48 h postoperatively C-SIA score at 6, 24, and 48 h postoperatively Static and dynamic pain scores at rest and movement in all aspects of the anterior and posterior hip, and surgical incision site in PACU, and at 6, 12, 24, and 48 h postoperatively Duration and incidence of sensory block the hip region TUG test at 48 h QMS test at 0 and 90 degrees at 12 h and 24 h Incidence of sleep disturbance Duration of hospitalization Patient satisfaction, nausea and vomiting, and scores for dizziness	PO: no intergroup difference in 24-h fentanyl consumption No intergroup differences in fentanyl consumption at any time interval, PCA requirements, postoperative pain scores, C-SIA score, TUG test, patient satisfaction, sleep disturbance, nausea/vomiting, dizziness, length of stay^a^ PENG block: improved QMS test at 90 degrees at 12 h (all other QMS values similar between the two groups) PENG block: fewer incidences of sensory loss in the hip region, and shorter duration of sensory loss	Despite the fact that both SIFIB and PENG block can anesthetize the articular branches of the femoral and accessory obturator nerves, this trial was powered to detect a 30% decrease in fentanyl consumption at 24 h with PENG block. Consequently, the trial may have been underpowered for the selected primary outcome
Zheng et al.^47^	70 total	PENG block: Ropivacaine 0.5%, 20 mL Sham: Saline 20 mL Both: Intra-articular injection with Ropivacaine 0.5%, 20 mL	Posterolateral	Preoperative (before general anesthesia)	General anesthesia	Quadriceps strength with handheld dynamometer (PACU, 6, 24, and 48 h)	Maximal VAS pain score at PACU stay	Cold sensation in the skin at 30 min and PACU Quadriceps strength in PACU, 6, 24, and 48 h Pain scores in PACU, 6, 24, and 48 h Opioid use at PACU, 6, 24, and 48 h Opioids-related side effects at PACU, 6, 24, and 48 h Satisfaction	PO: the highest pain scores in the PACU were significantly different in favor of PENG block (mean difference of 1.9 (0.5–3.3)) No significant between-group differences in quadriceps strength levels and in the incidence of decreased skin sensation^a^ No differences in the two groups’ pain scores at rest during the study period^a^ The two groups had similar levels of rescue opioid use during their PACU stay and 48 h after PACU discharge^a^ Reduced incidence of vomiting in PENG block No difference in satisfaction^a^	Inclusion criteria was elective THA, however; 6 and 8 fractures were included in the placebo and PENG groups, respectively

C-SIA: comprehensive Silverman integrated approach; ESP: erector spinae plane; HOOS JR: Hip Disability and Osteoarthritis Outcome Score for joint replacement; LFCN: lateral femoral cutaneous nerve; NRS: numeric rating score; PACU: postanesthetic care unit; PAI: periarticular local anesthetic infiltration; PCA: patient-controlled analgesia; PENG: pericapsular nerve group; PO: primary outcome; PROMIS-10: patient-reported outcome measures information system; SIFIB: suprainguinal fascia iliaca block; THA: total hip arthroplasty; TUG: timed up and go test; QMS: Quadriceps muscle strength; QLB: quadratus lumborum block; QoR-40: quality of recovery-40; TFPB: transversalis fascial plane block VAS: visual analog scale.

a The sample size was not powered to detect a difference in these secondary outcomes. Therefore, the lack of observed differences could be due to an underpowered sample size.

**Table 3.** Analgesic alternatives to PENG block for THA.

	Static pain	Dynamic pain	Breakthrough opioid consumption	Quadriceps paralysis	Side effects	Comments
Intrathecal Morphine vs. PENG block^39^	No intergroup differences in maximum pain over 48 h	No intergroup differences in maximum pain over 48 h	No intergroup differences in cumulative consumption over 48 h	No intergroup differences in failure of straight leg raise	No intergroup differences in nausea/vomiting/pruritus/blood pressure	
IIFIB vs. PENG block^40^	PENG block: less static pain over 48 h	PENG block: less dynamic pain over 48 h	PENG block: less opioid consumption	PENG block: less residual weakness at 6 h PENG block: less residual weakness at 24 h and 48 h No intergroup differences in time to ambulation	PENG block: less nausea/vomiting	PENG block group also received LFCN block
SIFIB vs. PENG Block^41^	No intergroup differences over 48 h	SIFIB: lower dynamic pain scores at 3 and 36 h	No intergroup differences in cumulative opioid consumption over 48 h	PENG block: less residual weakness at 3 h and 6 h	No intergroup differences in nausea/vomiting/pruritus	
Anterior QLB vs. PENG Block^44^	No intergroup differences over 48 h	No intergroup differences over 48 h	No intergroup differences over 48 h	Decreased quadricep strength at 3 h with anterior QLB vs. PENG block Earlier mobilization with PENG block compared to anterior QLB	No intergroup differences in nausea/vomiting/pruritus/urinary retention	
Lateral QLB vs. PENG Block^43^	No intergroup differences over 72 h	Lateral QLB: lower dynamic pain scores over 72 h	PENG block consumed more opioids at 36 h, 48 h, 60 h, and 72 h	No intergroup differences in time to ambulation, rate of same-day discharge	No intergroup differences in nausea/pruritus	Both groups also received PAI. PENG block group also received LFCN block
L4 ESP Block vs. PENG Block^45^	No intergroup differences over 48 h	No intergroup differences over 48 h	No intergroup differences over 48 h	No intergroup differences during first 24 h after surgery	No intergroup differences in nausea/orthostatic hypotension/return of bowel function/chronic pain	
PAI vs. PENG Block^46^	PAI: lower static pain scores over 48 h	PAI: lower dynamic pain scores at 3 and 6 h	No intergroup differences over 48 h	No intergroup differences over 48 h	No intergroup differences in nausea/vomiting/pruritus/urinary retention	Robust PAI regimen that encompassed both the anterior and posterior capsules of the hip joint
PAI vs. PAI + PENG Block^48^	No intergroup differences in worst postoperative pain over 48 h	Not assessed	No intergroup differences over 48 h	No intergroup differences in TUG test and isometric strength of adductor muscles (POD1)	No intergroup differences in nausea/vomiting/pruritus	

ESP: erector spinae plane; IIFIB: infrainguinal fascia iliaca block; LFCN: lateral femoral cutaneous nerve; PAI: periarticular infiltration; PENG: pericapsular nerve group; POD: postoperative day; QLB: quadratus lumborum block; SIFIB: suprainguinal fascia iliaca block; THA: total hip arthroplasty; TUG: timed up and go.

For any given alternative to PENG block, if more than one trial existed, only the highest-quality study is summarized in Table 3.

### PENG block versus intrathecal morphine

In 2025, Oremus et al.^39^ randomized 60 patients undergoing THA (with spinal anesthesia) to receive intrathecal morphine (100 µg) or PENG block (using 20 mL of levobupivacaine 0.5% and 2 mg of dexamethasone). These authors found that, compared with intrathecal morphine, PENG block resulted in noninferior maximum pain at rest, maximum pain with hip flexion, and cumulative breakthrough morphine consumption over 48 h. Furthermore, age-adjusted straight leg raise test failure rates were similar between the two groups.^39^

### PENG block versus alternative nerve blocks

In the literature, PENG block has been compared with four different blocks for THA: infrainguinal fascia iliaca block, suprainguinal fascia iliaca block (SIFIB), quadratus lumborum block (QLB), and lumbar erector spinae plane (ESP) block.

In 2025, Vetrone et al.^40^ compared PENG block (using 20 mL of ropivacaine 0.5%) combined with lateral femoral cutaneous nerve (LFCN) block (using 10 mL of ropivacaine 0.5%) with infrainguinal fascia iliaca block (using 20 mL of ropivacaine 0.5%). These authors reported lower pain at rest (6, 24, and 48 h), lower dynamic pain (6, 24, and 48 h), decreased breakthrough opioid consumption, greater muscle strength, and improved hip flexion (6, 24, and 48 h) with PENG block.^40^

To date, two randomized trials have compared PENG block with SIFIB.^41,42^ In the first trial, Aliste et al.^41^ randomized 40 patients undergoing THA to postoperative PENG block (using 20 mL of epinephrine-containing levobupivacaine 0.5%) or SIFIB (using 40 mL of epinephrine-containing levobupivacaine 0.25%). These authors observed no intergroup differences in pain scores, opioid consumption, or hospital length of stay. Despite similar abilities to participate in physiotherapy in the two study groups, PENG block resulted in lower incidences of quadriceps motor block at 3 h (45% vs. 90%; p < 0.001) and 6 h (25% vs. 85%; p < 0.001) as well as better preservation of hip adduction at 3 h (p = 0.023). In the second trial, Vichainarong et al.^42^ randomized 60 patients undergoing THA to preoperative PENG block or SIFIB (performed after induction of general anesthesia). Although SIFIB inherently blocks the femoral and accessory obturator nerves, as well as their articular branches, these authors inexplicably powered the study as a superiority trial in order to detect a 30% decrease in fentanyl consumption at 24 h with PENG block. Consequently, the trial was unable to detect any intergroup differences in terms of opioid consumption, pain scores, patient satisfaction, sleep disturbance, or opioid-related adverse events. However, patients receiving PENG block demonstrated improved quadriceps strength at 12 h and decreased and shorter sensory blockade in the hip region.^42^

To date, two randomized trials have compared PENG block with QLB for THA.^43,44^ In the first trial, Hay et al.^43^ randomized 101 patients to lateral QLB (30 mL of ropivacaine 0.25%) or combined PENG block (20 mL of ropivacaine 0.25%) and LFCN block (10 mL of ropivacaine 0.25%). In addition, all study participants received periarticular local anesthetic infiltration (PAI) using 1 mL/kg of a solution containing ropivacaine 0.25%, epinephrine, clonidine, and ketorolac. The injection sites for PAI were not explicitly specified. Hay et al.^43^ found no intergroup differences in terms of pain at rest, time to first ambulation, distance ambulated, opioid-related adverse effects, or hospital length of stay. Moreover, functional outcome measures using the Hip Disability and Osteoarthritis Outcome Score (HOOS) and Patient-Reported Outcome Measures Information System (PROMIS) physical and mental scores were similar. However, compared with PENG block, lateral QLB resulted in less pain with movement and was associated with lower opioid consumption at 36, 48, 60, and 72 h (all p ≤ 0.04).^43^ In the second trial (n = 60), Et et al.^44^ compared PENG block (using 20 mL of bupivacaine 0.5%) with anterior QLB (using 30 mL of bupivacaine 0.5%). No PAI was employed. Postoperatively, no intergroup differences were observed in static or dynamic pain scores or opioid consumption during the first 48 h. However, PENG block resulted in improved quadriceps strength at 3 h (23.3% vs. 63%; p = 0.019) and a shorter time to first mobilization (p = 0.011).^44^

In 2025, Buffoli et al.^43^ compared PENG block (using 20 mL of ropivacaine 0.5%) combined with LFCN block (using 10 mL of ropivacaine 0.5%) with L4 ESP block (using 30 mL of ropivacaine 0.5%) in 62 patients undergoing THA. These authors observed no intergroup differences in terms of static or dynamic pain scores (0–48 h) or cumulative opioid consumption (48 h). Although the ESP group displayed a higher incidence of motor block (16% vs. 3.2%), this difference did not reach statistical significance.

In summary, the best available evidence suggests that PENG block is superior to infrainguinal fascia iliaca block. Although PENG block provides similar analgesia and breakthrough opioid consumption compared with SIFIB, anterior QLB, or L4 ESP block, it may improve motor strength during the initial postoperative period, thereby facilitating early patient mobilization. In the setting of PAI, lateral QLB and combined PENG–LFCN block provide similar pain control and functional outcomes (including ambulation). However, lateral QLB may be associated with lower dynamic pain and reduced opioid requirements (from 36 to 72 h).

### PENG block versus PAI

Because PAI is commonly used for THA, a series of randomized trials sought to investigate the benefits of PENG block either as an alternative or as an adjunct to PAI.

**Figure 3.**
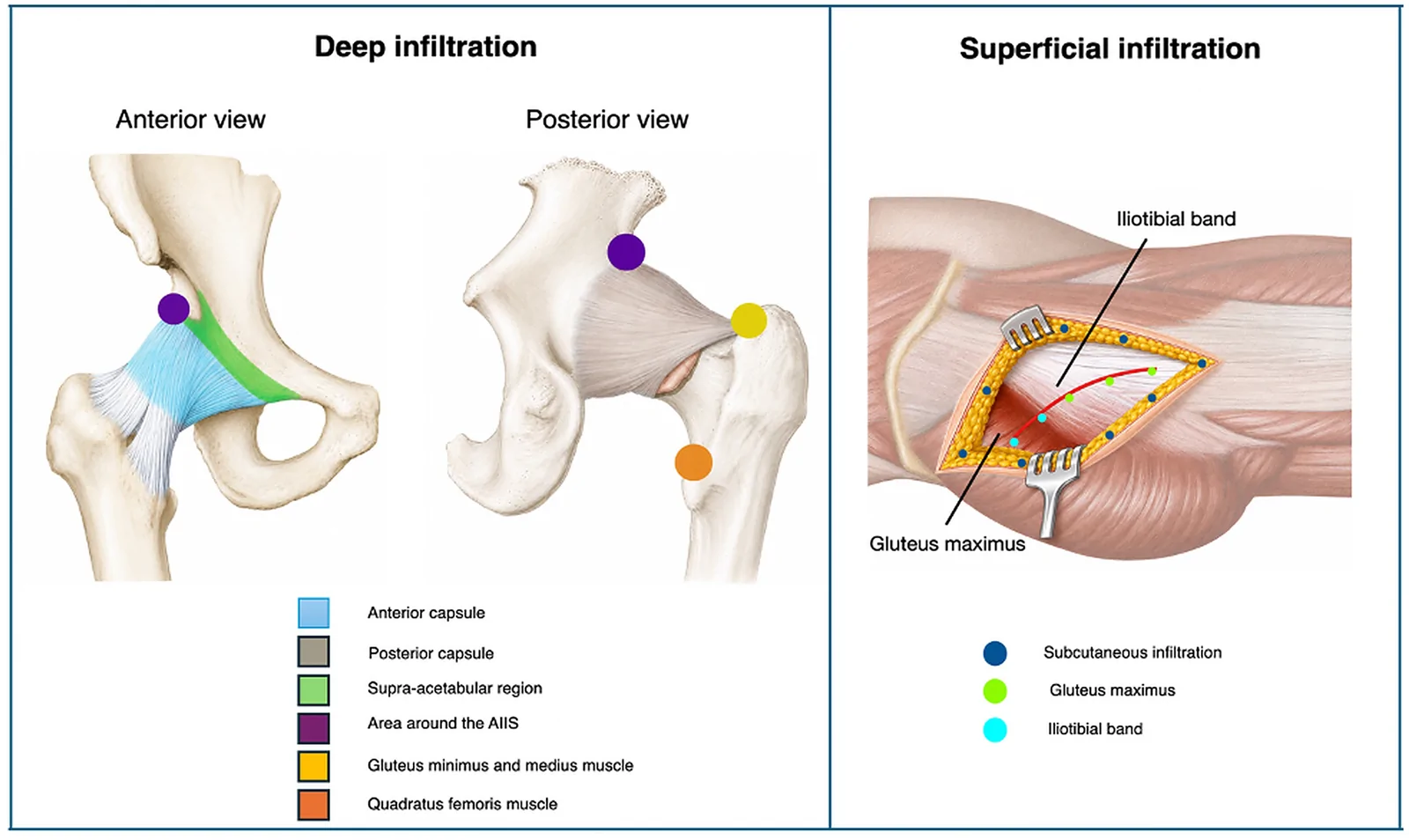
The admixture (60 mL of 0.25% bupivacaine with epinephrine 5 µg/mL and 30 mg of ketorolac) was prepared in two 30 mL syringes at the start of surgery. After insertion of the acetabular component and before insertion of the femoral stem, the surgeon infiltrated the deep tissues with the first 30 mL syringe, taking care to avoid the deep external rotator muscle group to prevent sciatic nerve block. The following structures were infiltrated: the area around the anterior inferior iliac spine (purple dot), the supra-acetabular region (green), the anterior capsule (light blue), the posterior capsule (light brown), the plane of the gluteus minimus and medius muscles (yellow dot), and the plane of the quadratus femoris muscle (orange dot). Before wound closure, the second 30 mL syringe was used to infiltrate the gluteus maximus muscle (cyan dots), the iliotibial band (green dots), and the subcutaneous tissues and skin (dark-blue dots).

In 2023, Bravo et al.^46^ compared PENG block (using 20 mL of adrenalized bupivacaine 0.5%) with PAI in 60 patients undergoing THA. For PAI, 30 mL of bupivacaine 0.25% (with epinephrine 5 μg/mL and 30 mg of ketorolac) was infiltrated into the deep tissues (i.e. anterior and posterior capsules, gluteus minimus and medius muscles, supraacetabular region, area around the anterior inferior iliac spine, and quadratus femoris muscle and avoided the deep hip external rotator muscle group in order to prevent sciatic nerve block) (Figure 3). Furthermore, before wound closure, the gluteus maximus muscle, iliotibial band, subcutaneous tissues, and skin were infiltrated with an additional 30 mL of the same local anesthetic (LA) admixture (Figure 3). Bravo et al.^46^ found that, compared with PENG block, PAI resulted in lower static pain scores (first 48 h) and lower dynamic pain scores (at 3 and 6 h) (all p < 0.05). However, there were no intergroup differences in terms of opioid requirements, motor weakness, physiotherapy performance, or hospital length of stay.

In stark contrast, in 2024, Et et al.^44^ reported lower dynamic pain scores at 3 and 6 h (p = 0.02 and p < 0.01, respectively) and a longer time to first opioid requirement with PENG block (using 20 mL of bupivacaine 0.5%) than with PAI (using 60 mL of bupivacaine 0.25%) (p = 0.009). However, in their trial, Et et al.^44^ administered only intra-articular LA, without infiltration elsewhere in the surgical field. Thus, the differences in PAI techniques may explain the discrepancy between the findings of Et et al. and Bravo et al.'s results.^46^

In parallel with the aforementioned studies comparing PENG block with PAI, two randomized trials investigated the benefits of adding PENG block as an adjunct to PAI.^47,48^ In 2022, Zheng et al.^47^ randomized 70 patients undergoing THA under general anesthesia and receiving intra-articular LA injection (20 mL of ropivacaine 0.5%) to PENG block (20 mL of ropivacaine 0.5%) or placebo. The highest pain scores in the postanesthesia care unit (PACU) were higher in the placebo group (5.2 (3.1) vs. 3.3 (2.7) on a 0–10 scale; p < 0.01). Furthermore, the control group required more intraoperative opioids (83.0 (29.5) vs. 69.1 (25.7) mg morphine; p = 0.04). However, there were no intergroup differences in terms of patient satisfaction, pain scores (other than the highest scores in the PACU), or postoperative opioid consumption in the PACU or at 48 h.

In 2024, Ferré et al.^48^ randomized 60 patients undergoing THA under general anesthesia and receiving PAI (80 mL of ropivacaine 0.2% distributed equally among the intra-articular, periarticular, intermuscular, and subcutaneous tissues) to PENG block (20 mL of ropivacaine 0.475%) or placebo. Both groups displayed similar pain scores, postoperative opioid requirements, incidences of opioid-related adverse effects, timed up and go (TUG) test, and levels of patient satisfaction. The difference between the findings of Ferré et al. and Zheng et al. may be attributable to the PAI regimen. Whereas Ferré et al. implemented a robust regimen (80 mL) targeting all potential sources of postoperative pain, Zheng et al. administered only 20 mL of intra-articular LA.

In summary, the LA and injection sites used for PAI varied across trials and may explain the discordant findings. However, the best available evidence suggests that, compared with PENG block, PAI results in lower dynamic pain scores during the first 6 h and lower static pain scores during the first 48 h. Furthermore, the addition of PENG block to a robust PAI regimen appears to provide minimal incremental benefit.

## Discussion

Currently, the best available evidence suggests that, from an analgesic standpoint, PENG block is noninferior to intrathecal morphine (100 µg) and superior to infrainguinal fascia iliaca block. Furthermore, PENG block provides an equianalgesic, motor-sparing alternative to SIFIB, anterior QLB, and possibly lumbar ESP block for THA. However, a rigorous PAI regimen may outperform PENG block.

The mechanisms underlying (femoral) motor block associated with the different nerve blocks used for THA require discussion. As expected, SIFIB results in femoral blockade because the main trunk of the femoral nerve travels within the fascia iliaca compartment. In the case of anterior QLB (which aims to deposit LA between the quadratus lumborum and psoas muscles), LA may migrate or be inadvertently deposited within the psoas muscle, thereby potentially reaching the roots of the lumbar plexus.^49^ Although PENG block is purported to avoid femoral nerve block, the available evidence supports a reduction but not complete avoidance. For example, Aliste et al.^41^ documented a 45% incidence of paresis/paralysis and a 65% incidence of sensory blockade of the anterior thigh at 3 h. Proposed explanations include LA injection into the iliopsoas muscle (thereby leading to LA diffusion through the muscle to the femoral nerve); LA injection medial to the iliopubic eminence (thereby leading to LA diffusion medial to the iliopsoas muscle toward the femoral nerve); or LA injection with an unnecessarily high volume.^50^ In a recent cadaveric study, Leurcharusmee et al.^51^ demonstrated that the volume required for a femoral-sparing PENG block in 90% of participants is 13.2 mL. Because most published trials have used >13.2 mL for PENG block, this latter mechanism may explain incidental femoral nerve block, even in the hands of experienced operators. Thus, future trials should investigate PENG block for THA using LA volumes <13 mL.^3^

The concept of motor-sparing nerve block (for THA) deserves special mention for two reasons. First, the purported success (or failure) of a motor-sparing nerve block cannot be dissociated from the objective measurement of motor blockade. In the literature, motor assessment includes both early direct evaluations (e.g. assessments at 3–6 h)^39–41,44,46,47^ and later indirect measures (e.g. the ability to participate in physiotherapy on postoperative day 1).^43,48^ This variability may hinder comparisons of the motor-sparing properties of PENG block across different trials. Second, the concept of motor sparing inherently depends on the timing of physiotherapy. For example, the benefit of completely avoiding muscle weakness is relevant only if early postoperative rehabilitation is feasible. In the absence of early physiotherapy, this advantage becomes less meaningful. Furthermore, even when early physiotherapy is implemented, one needs to define “early” (and more specifically “how early”). For example, if the first session of physiotherapy takes place >3 h after surgery, perhaps, the 45% incidence of quadriceps paralysis/paresis observed with PENG block (at 3 h) becomes a lesser concern.^41^ Consequently, further investigation is required to determine the optimal method for assessing motor block as well as the optimal timing of early physiotherapy (Table 4).

**Table 4.** Possible areas for future research regarding PENG block (and PAI) for THA.

Motor-sparing effect and analgesia for PENG block with injectate <13 mL
Benefits of perineural adjuncts (e.g. dexamethasone and dexmedetomidine)
Benefits of PENG block for different surgical approaches (i.e. posterolateral vs. lateral vs. anterior) for THA.
Optimal block timing for PENG block (i.e. preoperative vs. postoperative)
Optimal technique (i.e. injection sites and volume) for motor-sparing PAI
Impact of PENG block in enhanced recovery protocols
Optimal method for assessment of motor block
Optimal schedule for early postoperative physiotherapy

PAI: periarticular local anesthetic infiltration; PENG: pericapsular nerve group; THA: total hip arthroplasty.

The role of PAI in THA also warrants discussion. Although the anterior hip capsule constitutes the primary source of nociception,^52^ the hip joint contains additional sources of pain.^53^ Therefore, a strategy such as PAI, which targets not only the anterior hip capsule but also the posterior aspect of the hip (innervated by the sacral plexus), may outperform PENG block, whose benefits are limited to the anterior hip capsule. Interestingly, PAI does not appear to eliminate the risk of motor weakness. In 2023, Bravo et al.^46^ reported a 33% incidence of quadriceps weakness (coupled with a 33% incidence of sensory blockade of the anterior thigh) at 3 h after PAI. These authors hypothesized that, because their PAI technique encompassed LA injection of the anterior capsule itself, LA deposited in this location could have diffused to the femoral nerve.^46^ This mechanism of action may be most relevant for posterolateral and lateral surgical approaches for THA, as the injection points are mostly blind. Consequently, future trials are required to determine the optimal technique (i.e., injection sites and LA volumes) for motor-sparing PAI (Table 4).

In the current review, only 10 randomized trials met the predetermined inclusion criteria. To date, most published trials either lack prospective registration or display undisclosed discrepancies between the registered and reported protocols (in terms of sample size and/or reported outcomes). Such discrepancies represent methodological shortcomings and may reflect investigator/reporting bias. Consequently, in an effort to publish only unbiased data, some journals have proposed adopting an automatic rejection policy when reported protocols differ from their registered counterparts.^54^ The fact that only 10 trials were retained may constitute a limitation in terms of generalizability. Nonetheless, this methodological approach was adopted to protect readers from investigator bias, publication bias, and potentially inaccurate information. Furthermore, the present review was not prospectively registered. Poststudy registration may have introduced bias to our results and represents a limitation of this study. Consequently, further randomized trials investigating PENG block are required: these trials should thrive for prospective registration and refrain from surreptitiously altering the research protocol after the start of patient recruitment (Table 4).

Several systematic reviews and meta-analyses evaluating PENG block for THA have recently been published.^55–58^ In contrast to our approach, these reviews pooled all available randomized trials regardless of registration integrity and combined structurally different comparators (e.g. sham, fascia iliaca block, QLB, periarticular infiltration, and intrathecal morphine) into single summary estimates. Their conclusions were generally favorable, with PENG block reported to reduce postoperative pain and opioid consumption compared with sham or no block and to improve functional recovery.^55,57^ A network meta-analysis identified the combination of PENG block and periarticular infiltration as the most effective strategy.^56^ However, most trials included in these analyses lacked prospective registration or displayed undisclosed discrepancies between the registered and reported protocols, raising concerns regarding investigator and publication bias. Our review differs in two important respects. First, we deliberately restricted inclusion to prospectively registered randomized trials without protocol discrepancies, thereby excluding 34 of 44 otherwise eligible trials in an effort to protect readers from biased estimates. Second, rather than aggregating heterogeneous comparators into a single pooled effect, we synthesized the evidence in a comparator-specific manner. As a result, our conclusions are more conservative and more nuanced than those of previous reviews. Although PENG block appears to be an equianalgesic, motor-sparing alternative to SIFIB, anterior QLB, and lumbar ESP block, the current evidence is insufficient to establish its role relative to intrathecal morphine or a robust periarticular infiltration regimen, both of which may provide comparable or superior analgesia. To the best of our knowledge, this is the first systematic review of PENG block for THA to require prospective registration and protocol integrity as prerequisites for study inclusion.

The clinical implications of these findings require a clear differentiation between inpatient and outpatient THA. For inpatients, the potential risks of respiratory depression and urinary retention associated with intrathecal morphine may be less problematic because patients are monitored on the surgical ward. Since intrathecal morphine is inherently motor sparing, it may represent an effective, low-cost alternative to PENG block, which is not completely free of motor blockade.^41^ For outpatients, intrathecal morphine becomes less desirable. In this setting, a robust PAI regimen could be used in lieu of PENG block because of lower static pain (first 48 h) and lower dynamic pain (first 6 h) in the postoperative period.^46^ Consequently, going forward, one needs to reassess the clinical indications for PENG block both in inpatient THA (where intrathecal morphine and PAI could be used) and outpatient THA (where PAI could be employed).

## Conclusion

The limited available evidence supports the use of PENG block as a motor-sparing alternative to SIFIB, anterior QLB, and, possibly, lumbar ESP block for THA. However, intrathecal morphine may provide analgesia comparable to that of PENG block. Furthermore, a robust PAI regimen may outperform PENG block. Consequently, future large, well-conducted trials, with proper prospective registration and without discrepancies between the registered and reported protocols, are required to clarify the benefits of PENG block (using LA volumes <13 mL) compared with intrathecal morphine (for inpatient THA) and PAI (for inpatient and outpatient THA).

## Supplemental Material

sj-docx-1-imr-10.1177_03000605261476867 - Supplemental material for Pericapsular nerve group block for total hip arthroplasty: A systematic review Supplemental material, sj-docx-1-imr-10.1177_03000605261476867 for Pericapsular nerve group block for total hip arthroplasty: A systematic review by Daniela Bravo, Hernán Arancibia, Maibelyn Manzanilla, Álvaro Jara, Samita Pirotesak, Julián Aliste and De Q Tran in Journal of International Medical Research

sj-docx-2-imr-10.1177_03000605261476867 - Supplemental material for Pericapsular nerve group block for total hip arthroplasty: A systematic review Supplemental material, sj-docx-2-imr-10.1177_03000605261476867 for Pericapsular nerve group block for total hip arthroplasty: A systematic review by Daniela Bravo, Hernán Arancibia, Maibelyn Manzanilla, Álvaro Jara, Samita Pirotesak, Julián Aliste and De Q Tran in Journal of International Medical Research

sj-pdf-3-imr-10.1177_03000605261476867 - Supplemental material for Pericapsular nerve group block for total hip arthroplasty: A systematic review Supplemental material, sj-pdf-3-imr-10.1177_03000605261476867 for Pericapsular nerve group block for total hip arthroplasty: A systematic review by Daniela Bravo, Hernán Arancibia, Maibelyn Manzanilla, Álvaro Jara, Samita Pirotesak, Julián Aliste and De Q Tran in Journal of International Medical Research
