# Supplementary material for: Pericapsular nerve group block for total hip arthroplasty: A systematic review
Source: J Int Med Res. 2026 Aug 25;54(8):03000605261476867. doi: 10.1177/03000605261476867 (PMC13519178; doi:10.1177/03000605261476867)
Supplement: Supplementary file 1 [file sj-pdf-3-imr-10.1177_03000605261476867.pdf]

International Platform of Registered Systematic Review and Meta-analysis Protocols

INPLASY

PERICAPSULAR NERVE GROUP (PENG) BLOCK FOR TOTAL HIP ARTHROPLASTY: A SYSTEMATIC REVIEW

INPLASY202670024

doi: 10.37766/inplasy2026.7.0024

Received: 9 July 2026

Published: 9 July 2026

Bravo, D; Arancibia, H; Manzanilla, M; Jara, A; Pirotesak, S; Aliste, J; Tran, DQ.

Corresponding author:
Julián Aliste

julian.aliste@mcgill.ca

Author Affiliation:
University of Chile.

ADMINISTRATIVE INFORMATION

Support - No support.

Review Stage at time of this submission - Completed but not published.

Conflicts of interest - None declared.

INPLASY registration number: INPLASY202670024

Amendments - This protocol was registered with the International Platform of Registered Systematic Review and Meta-Analysis Protocols (INPLASY) on 9 July 2026 and was last updated on 9 July 2026.

INTRODUCTION

Review question / Objective The aim of this systematic review is to determine the analgesic and motor-sparing benefits of pericapsular nerve group (PENG) block in adult patients undergoing total hip arthroplasty.

Rationale Since its description in 2018, PENG block has expanded from hip fracture surgery to total hip arthroplasty (THA), where preservation of quadriceps function is paramount for early rehabilitation and ambulation. Multiple randomized trials have since investigated its analgesic benefits in this setting. However, the majority of these trials display methodological irregularities, such as retrospective trial registration, or potential investigator bias, such as undisclosed changes in primary outcome or sample size after the start of patient recruitment. These shortcomings result in a prohibitive knowledge gap.
Several systematic reviews and meta-analyses of PENG block for THA have recently been published. However, these reviews pooled all available randomized trials irrespective of the integrity of their registration, and combined structurally different comparators (sham, fascia iliaca block, quadratus lumborum block, periarticular local anesthetic infiltration, and intrathecal morphine) into single summary estimates. Their favorable conclusions may therefore be inflated by investigator and publication bias, and obscure comparator-specific differences that are clinically relevant.
A new systematic review is warranted in order to summarize the best evidence available while protecting readers from biased estimates. The present review differs from its predecessors in two fundamental respects: inclusion is restricted to prospectively registered randomized trials without discrepancies between registered and reported protocols, and the evidence is synthesized in a comparator-specific manner rather than aggregated into a single pooled effect. To our knowledge, this is the first systematic review of PENG block for THA to apply registration and protocol-integrity criteria as a prerequisite for inclusion.

Bravo et al. INPLASY protocol 202670024. doi:10.37766/inplasy2026.7.0024 Downloaded from https://inplasy.com/inplasy-2026-7-0024 /

Condition being studied Postoperative pain following total hip arthroplasty (THA). THA is one of the most frequently performed orthopedic procedures worldwide and is associated with moderate-to-severe postoperative pain, particularly during the first 48 hours and with movement. Inadequate analgesia delays mobilization, prolongs hospital length of stay, and increases opioid consumption and opioid-related adverse effects. Conversely, analgesic strategies that produce motor blockade of the quadriceps muscle may impair early physiotherapy and ambulation, which are central to enhanced recovery pathways and to same-day discharge protocols in outpatient THA.

The clinical challenge therefore lies in achieving effective analgesia while preserving motor function. Regional anesthetic techniques evaluated for this purpose include pericapsular nerve group (PENG) block, infrainguinal and suprainguinal fascia iliaca block, anterior quadratus lumborum block, lumbar erector spinae plane block, periarticular local anesthetic infiltration, and intrathecal morphine. The condition being studied is thus acute postoperative pain and early functional recovery in adult patients undergoing primary total hip arthroplasty.

METHODS

Search strategy The search was conducted from database inception until the first week of May 2026. Identical free-text search strings were applied across all four databases; no controlled vocabulary (MeSH/Emtree) terms or database-specific filters were used beyond the language and study-design restrictions described below.

Primary search string (MEDLINE, EMBASE, CINAHL, Google Scholar):

"pericapsular nerve group block" AND "hip arthroplasty"

Secondary search string, applied for completeness (all four databases):

"PENG block" AND "hip surgery"

Results were restricted to English-language randomized trials comparing PENG block with sham, no block, or an alternative analgesic technique. Titles and abstracts were screened by six coauthors, and the reference lists of all trials retrieved through the electronic searches were subsequently examined to identify additional randomized trials.

Participant or population Adult patients (≥18 years) undergoing primary total hip arthroplasty for any indication, under general or spinal anesthesia, with no restriction based on sex, ethnicity, surgical approach (anterior, lateral, or posterolateral), body mass index, or ASA physical status. Both inpatient and outpatient (ambulatory) total hip arthroplasty are eligible.

Ineligible participants. Patients undergoing hip surgery for indications other than total hip arthroplasty are excluded, specifically hip fracture repair and hip arthroscopy. Patients undergoing revision hip arthroplasty are not addressed. Trials enrolling mixed surgical populations (e.g., hip fracture repair together with total hip arthroplasty) are eligible only if results are reported separately for the total hip arthroplasty subgroup; otherwise they are excluded.

Intervention Ultrasound-guided pericapsular nerve group (PENG) block, performed preoperatively, intraoperatively, or postoperatively, using any local anesthetic agent, concentration, or volume, with or without adjuvants (e.g., dexamethasone, epinephrine). Single-injection and continuous catheter techniques are both eligible.

PENG block administered in combination with a lateral femoral cutaneous nerve (LFCN) block, or as an adjunct to periarticular local anesthetic infiltration, is also eligible, provided that the co-intervention is not administered to the comparator group in a manner that precludes attribution of the observed effect to PENG block.

Ineligible interventions. Trials in which PENG block is administered to all study groups and to all patients (i.e., where PENG block is not the variable under investigation) are excluded.

Comparator Any of the following, administered in lieu of PENG block:

Sham block, no block, or systemic analgesia alone (i.e., a multimodal oral or intravenous regimen without a regional technique); neuraxial analgesia, including intrathecal morphine; infrainguinal fascia iliaca block; suprainguinal fascia iliaca block; anterior or lateral quadratus lumborum block; lumbar (L4) erector spinae plane block; and periarticular local anesthetic infiltration, whether intra-articular only or as a comprehensive regimen targeting the joint capsule, periarticular tissues, intermuscular planes, and subcutaneous tissues.

Comparators are analyzed separately rather than pooled, since these techniques differ substantially in anatomical target, mechanism of action, and expected motor effects. Trials comparing PENG block with a comparator group that also receives PENG block are excluded.

Study designs to be included Randomized controlled trials only. Both parallel-group and, where applicable, crossover randomized designs are eligible; non-inferiority and superiority designs are both accepted.To be retained, a randomized

Bravo et al. INPLASY protocol 202670024. doi:10.37766/inplasy2026.7.0024 Downloaded from https://inplasy.com/inplasy-2026-7-0024 /

trial must additionally satisfy three methodological requirements: prospective registration in a recognized clinical trial registry prior to the start of patient recruitment; blinded outcome assessment; and the absence of discrepancies between the registered and the published protocol, in terms of sample size, primary outcome, or reported outcomes.Ineligible designs. Non-randomized studi.

Eligibility criteria Inclusion criteria (PICOS).
Population: adult patients (≥18 years) undergoing primary total hip arthroplasty.
Intervention: ultrasound-guided pericapsular nerve group (PENG) block, alone or in combination with a lateral femoral cutaneous nerve block, or as an adjunct to periarticular local anesthetic infiltration.
Comparator: sham block, no block or systemic analgesia alone; neuraxial analgesia including intrathecal morphine; an alternative regional technique (infrainguinal or suprainguinal fascia iliaca block, anterior or lateral quadratus lumborum block, lumbar erector spinae plane block); or periarticular local anesthetic infiltration.
Outcomes: at least one of static pain, dynamic pain, breakthrough opioid consumption, or quadriceps motor function.
Study design: randomized controlled trial.
Additional inclusion criteria beyond PICOS, with justification.
Prospective registration in a recognized clinical trial registry prior to the start of patient recruitment; blinded outcome assessment; and no discrepancy between the registered and the reported protocol with respect to sample size, primary outcome, or reported outcomes. These criteria are applied because retrospective registration and undisclosed protocol deviations are established sources of investigator and reporting bias, and their presence undermines confidence in the reported effect estimates.
Publication in English. This restriction is imposed because the review team cannot reliably verify the correspondence between registered and reported protocols in languages it does not read, which is central to the inclusion criteria above.
Exclusion criteria.
Surgical settings other than primary total hip arthroplasty (hip fracture repair, hip arthroscopy, revision arthroplasty); trials in which PENG block is administered to all study groups and to all patients; trials enrolling mixed surgical populations without separate reporting of the total hip arthroplasty subgroup; non-randomized designs; and randomized trials failing any of the three methodological requirements stated above.
Eligibility criteria are applied by six coauthors independently; disagreements are resolved by the senior author. Any post-registration modification to the eligibility criteria will be reported explicitly as a difference between the protocol and the review.

Information sources Bibliographic databases. MEDLINE, EMBASE, CINAHL, and Google Scholar, each searched from inception until the first week of May 2026. The search interfaces used were [confirmar: PubMed u Ovid para MEDLINE; Ovid o Elsevier para EMBASE; EBSCOhost para CINAHL].
Supplementary sources. The reference lists of all randomized trials retrieved through the electronic searches were hand-searched to identify additional eligible trials.
Trial registries. For every trial passing full-text screening, the corresponding registry record (e.g., ClinicalTrials.gov, Chinese Clinical Trial Registry, Clinical Trials Registry–India, EU Clinical Trials Register, or other registry cited by the authors) was retrieved and compared against the published report, in order to verify prospective registration and to detect discrepancies in sample size, primary outcome, or reported outcomes. Registries were consulted for verification of protocol integrity, not as a source of records for screening.
Sources not searched. No search of grey literature, dissertation and thesis databases, conference proceedings, or preprint servers was performed. Trial registries were not searched to identify unpublished or ongoing studies.

Main outcome(s) Postoperative pain intensity, assessed separately at rest (static pain) and on movement (dynamic pain, typically hip flexion or straight leg raise), measured on a numeric rating scale or visual analog scale (0–10 or 0–100). Outcomes are extracted at the time points reported by the primary trials, with particular attention to the early postoperative period (3, 6, 12, 24, and 48 hours), since this is the interval during which regional analgesia and early mobilization interact.
Where trials report a composite or maximum pain score over a defined interval, that measure is extracted as reported and identified as such.

Additional outcome(s) Breakthrough opioid consumption over the postoperative period, at the time points reported by the primary trials.
Quadriceps motor function, assessed either by direct early evaluation (quadriceps strength, straight leg raise test, incidence of quadriceps paresis or paralysis at 3 and 6 hours) or by late indirect measures (ability to perform physiotherapy on postoperative day 1). The two categories are reported separately and not combined, since the timing and method of motor assessment vary

Bravo et al. INPLASY protocol 202670024. doi:10.37766/inplasy2026.7.0024 Downloaded from https://inplasy.com/inplasy-2026-7-0024 /

substantially between trials and this variability is itself a finding of the review.

Side effects, including opioid-related adverse effects.

Quadriceps motor function is reported as an additional outcome of primary clinical interest rather than as an adverse event, since the clinical rationale for PENG block rests on the claim that it provides analgesia while sparing motor function.

Outcomes reported by individual trials but not pre-specified as extraction variables (e.g., hospital length of stay, timed up-and-go test, patient satisfaction) are described narratively where available, and are not synthesized across trials.

Data management Records retrieved from the four databases are exported and deduplicated, after which non-randomized studies are removed. Titles and abstracts are screened against the eligibility criteria, and the remaining records undergo full-text assessment.

Study selection is performed by six coauthors (DB, HA, MM, AJ, SP, JA) working independently. Data extraction is likewise performed independently by the same six coauthors. Any disagreement at either stage is resolved by the senior author (DQT), who acts as arbiter.

The pre-specified extraction variables are: static pain scores, dynamic pain scores, breakthrough opioid consumption, quadriceps paralysis or paresis, and side effects, each recorded at the time points reported by the primary trials. Trial characteristics are additionally tabulated: first author, year, sample size, study groups, surgical approach, block timing, type of anesthesia, method and timing of motor block assessment, primary and secondary outcomes, and main results.

For each trial passing full-text screening, the corresponding registry record is retrieved and compared against the published report to verify prospective registration and to identify discrepancies in sample size, primary outcome, or reported outcomes. Reasons for exclusion are recorded for every trial excluded at this stage and are reported in an appendix.

No systematic review software (e.g., Rayyan, Covidence) or automation tool was used. Screening and data extraction were performed manually by the six coauthors, and extracted data were compiled in a shared spreadsheet.

Quality assessment / Risk of bias analysis Risk of bias is assessed with the Cochrane risk-of-bias tool by two coauthors (DB, HA) working independently. Six domains are evaluated: adequacy of sequence generation; allocation concealment; blinding; handling of incomplete outcome data; selective outcome reporting; and other sources of bias, including study design issues, early trial termination, and baseline imbalance between study groups.

Each domain is categorized as low risk ("yes", green), high risk ("no", red), or unclear risk ("unclear", yellow). Disagreements between the two assessors are settled by the senior author (DQT). Results are presented as a risk-of-bias summary figure.

Because the eligibility criteria already impose stringent methodological requirements (prospective registration, blinded assessment, and protocol integrity), no further exclusion is planned in the event of high risk of bias in any single domain; the risk-of-bias assessment is used to characterize the retained evidence rather than to filter it.

Certainty of evidence. A formal GRADE assessment is not planned. Because the review does not perform quantitative pooling and the comparators are structurally heterogeneous, certainty in the body of evidence is appraised narratively, with explicit discussion of the limitations imposed by the small number of retained trials, the diversity of comparators, and the variability in the timing and method of motor assessment.

Strategy of data synthesis The synthesis is qualitative and comparator-specific. No quantitative meta-analysis is performed.

Trials are grouped according to the comparator against which PENG block is evaluated: intrathecal morphine; alternative nerve blocks (infrainguinal fascia iliaca block, suprainguinal fascia iliaca block, quadratus lumborum block, lumbar erector spinae plane block); periarticular local anesthetic infiltration; and PENG block as an adjunct to periarticular local anesthetic infiltration. Within each group, findings for static pain, dynamic pain, breakthrough opioid consumption, quadriceps motor function, and side effects are described narratively and tabulated, and the direction and consistency of effect across trials is assessed. Results are then integrated into an overall synthesis that distinguishes comparator-specific conclusions from the residual uncertainty that applies to the evidence base as a whole.

Rationale for not performing a meta-analysis. Pooling was judged inappropriate for three reasons. First, the comparators are structurally heterogeneous: they differ in anatomical target, mechanism of action, and expected motor effects, such that a single summary estimate of "PENG block versus other techniques" would not correspond to any answerable clinical question. Second, the local anesthetic regimens differ

Bravo et al. INPLASY protocol 202670024. doi:10.37766/inplasy2026.7.0024 Downloaded from https://inplasy.com/inplasy-2026-7-0024 /

substantially between trials, particularly for periarticular infiltration, where volume and injection sites range from intra-articular injection alone to comprehensive multi-site regimens; these differences are themselves a plausible explanation for discordant findings. Third, the timing and method of motor assessment vary between trials, from direct evaluation at 3 to 6 hours to indirect assessment of physiotherapy performance on postoperative day 1, which precludes meaningful pooling of the motor-sparing outcome.
Effect measures, statistical model, heterogeneity, missing data. Not applicable, as no quantitative synthesis is undertaken. Between-trial differences are explored narratively rather than through statistical tests of heterogeneity. No imputation of missing data is performed; outcomes are reported as published, and the absence of a given outcome in a given trial is stated explicitly.
Should the number of methodologically eligible trials sharing a common comparator and a common outcome definition increase in future updates of this review, a quantitative synthesis restricted to that comparator may be considered.

Subgroup analysis Not applicable. No quantitative synthesis is undertaken, and therefore no statistical subgroup analysis is planned. Trials are, however, stratified a priori by comparator, as described in item 22; this stratification is structural to the synthesis rather than a subgroup analysis of a pooled estimate.

Sensitivity analysis Not applicable. No quantitative synthesis is undertaken. The restriction of inclusion to prospectively registered trials without protocol discrepancies is applied as an eligibility criterion rather than explored through sensitivity analysis; consequently, no analysis including or excluding trials on the basis of methodological quality is planned.

Language restriction Only randomized trials published in English are considered for inclusion. This restriction is imposed because verification of the correspondence between registered and reported protocols, which is central to the eligibility.

Country(ies) involved Chile, Canadá, Thailand.

Keywords Countries involved: Chile; Canada; ThailandAfiliaciones por autor:Daniela Bravo — Department of Anesthesiology and Perioperative Medicine, Hospital Clínico Universidad.

Contributions of each author
Author 1 - Daniela Bravo.
Email: dbravoadvis@uchile.cl
Author 2 - Hernan Arancibia.
Author 3 - Maibelyn Manzanilla.
Author 4 - Álvaro Jara.
Author 5 - Samita Pirotesak.
Author 6 - Julián Aliste.
Email: julian.aliste@mcgill.ca
Author 7 - De Tran.

Bravo et al. INPLASY protocol 202670024. doi:10.37766/inplasy2026.7.0024 Downloaded from https://inplasy.com/inplasy-2026-7-0024 /
