# Supplementary material for: Pericapsular nerve group block for total hip arthroplasty: A systematic review
Source: J Int Med Res. 2026 Aug 25;54(8):03000605261476867. doi: 10.1177/03000605261476867 (PMC13519178; doi:10.1177/03000605261476867)
Supplement: Supplementary file 2 [file sj-docx-1-imr-10.1177_03000605261476867.docx]

**Appendix A**. Discarded studies investigating PENG block for THA.

| **TRIAL** | **REASON FOR EXCLUSION** |
| --- | --- |
| Abd El-Gawad et al (2025) **[19]** | No trial registration |
| Adbelrahman et al (2025) **[20]** | Retrospective registration |
| Abdelsalam et al (2024) **[21]** | No trial registration |
| Abdou et al (2024) **[22]** | No trial registration |
| Carella et al (2023) **[23]** | Discrepancy between registered sample size, calculated sample size (in manuscript), and number of randomized cases  Discrepancy between registered and reported secondary outcomes |
| Cho et al (2025) **[24]** | Discrepancy between registered and reported primary outcome  Discrepancy between registered and reported secondary outcomes |
| Choi et al (2022) **[25]** | Discrepancy between registered and reported secondary outcomes |
| Chung et al (2022) **[26]** | Registration incomplete (no reported period of patient recruitment)  Discrepancy between registered and reported secondary outcomes |
| Cui et al (2024) **[27]** | Discrepancy between registered and reported secondary outcomes |
| Domagalska et al. (2023) **[28]** | Retrospective registration |
| Duan et al. (2023) **[29]** | Discrepancy between registered and reported intervention groups and techniques |
| Elnefiawy et al (2023) **[30]** | Discrepancy between registered sample size, calculated sample size (in manuscript), and number of randomized cases  Discrepancy between registered and reported secondary outcomes |
| Gonabal et al (2023) **[31]** | Retrospective registration |
| Hanauer et al (2025) **[32]** | Discrepancy between registered sample size, calculated sample size (in manuscript), and number of randomized cases |
| Hu et al (2022) **[33]** | Sham PENG block not included in the registry  Discrepancy between registered and reported secondary outcomes |
| Hu et al (2025) **[34]** | Discrepancy between registered and reported secondary outcomes |
| Iglesias et al (2022) **[35]** | No trial registration |
| Kalashetty et al (2022) **[36]** | Retrospective registration |
| Kamel et al (2024) **[37]** | No trial registration |
| Kukreja et al (2023) **[38]** | Discrepancy between registered sample size, calculated sample size (in manuscript), and number of randomized cases  Changes in secondary outcomes after trial initiation |
| Kücúk et al (2024) **[39]** | Retrospective registration |
| Lee et al (2024) **[40]** | Discrepancy between registered and reported sample sizes  Change in primary outcome after trial initiation  Discrepancy between registered and reported secondary outcomes |
| Liang et al (2023) **[41]** | Discrepancy between registered and reported sample sizes  Discrepancy between registered and reported primary outcome  Discrepancy between registered and reported secondary outcomes |
| Lin et al (2022) **[42]** | Discrepancy between registered and reported sample sizes |
| Medhat et al (2023) **[43]** | Retrospective registration |
| Pascarella et al (2021) **[44]** | Status changed from "non-randomized" to "randomized" after the end of patient recruitment. |
| Svraka et al (2024) **[45]** | No trial registration |
| Turkan (2024) **[46]** | Retrospective registration |
| Vamshi et al (2023) **[47]** | Discrepancy between registered and reported secondary outcomes |
| Wang et al (2023) **[48]** | Retrospective registration |
| Ye et al (2023) **[49]** | Retrospective registration |
| Zheng et al (2021) **[50]** | Retrospective registration |
| Zhu et al (2024) **[51]** | Discrepancy between registered and reported patient recruitment periods; according to registry, patient enrolment predates registration  Discrepancy between registered and reported secondary outcomes |
| Erdem et al (2026) **[52]** | Change in definition of the primary outcome after the start of patient recruitment  Change in sample size after the end of patient recruitment |

PENG = pericapsular nerve group; THA = total hip arthroplasty.
