# Supplementary material for: Pericapsular nerve group block for total hip arthroplasty: A systematic review
Source: J Int Med Res. 2026 Aug 25;54(8):03000605261476867. doi: 10.1177/03000605261476867 (PMC13519178; doi:10.1177/03000605261476867)
Supplement: Supplementary file 3 [file sj-docx-2-imr-10.1177_03000605261476867.docx]

**PRISMA 2020 Checklist**

*PENG block for total hip arthroplasty: a systematic review (JIMR-26-1683)*

| **Section / Topic** | **Item #** | **Checklist item** | **Location where item is reported** |
| --- | --- | --- | --- |
| **TITLE** | | | |
| Title | 1 | Identify the report as a systematic review. | Title page — “…A Systematic Review” |
| **ABSTRACT** | | | |
| Abstract | 2 | See the PRISMA 2020 for Abstracts checklist (structured: Objective, Methods, Results, Conclusion). | 1-Abstract |
| **INTRODUCTION** | | | |
| Rationale | 3 | Describe the rationale for the review in the context of existing knowledge. | 3-Introduction, para. 1–2 |
| Objectives | 4 | Provide an explicit statement of the objective(s) or question(s) the review addresses. | 3-Introduction, last para.; 1-Abstract – Objective |
| **METHODS** | | | |
| Eligibility criteria | 5 | Specify the inclusion and exclusion criteria and how studies were grouped for the synthesis. | 4-Methods — prospectively registered RCTs, English, no protocol discrepancies; grouped by comparator |
| Information sources | 6 | Specify all databases, registers, etc., searched and the date each was last searched. | 4-Methods — MEDLINE, EMBASE, CINAHL, Google Scholar (inception – first week of May 2026) |
| Search strategy | 7 | Present the full search strategies for all databases, including any filters and limits used. | 4-Methods — search terms (“pericapsular nerve group block”, “hip arthroplasty”, alternates); English-language RCT filter |
| Selection process | 8 | Specify the methods used to decide whether a study met the inclusion criteria, including how many reviewers screened. | 4-Methods — six co-authors screened; disagreements resolved by senior author (DQT) |
| Data collection process | 9 | Specify the methods used to collect data from reports, including how many reviewers collected data. | 4-Methods — six co-authors independently extracted data; disagreements resolved by DQT |
| Data items | 10a | List and define all outcomes for which data were sought. | 5-Methods — static/dynamic pain, breakthrough opioid consumption, quadriceps paralysis, side effects |
| Data items | 10b | List and define all other variables for which data were sought. | 5- Methods; Tables 1–2 — block technique, LA type/volume/dose, comparator, anesthesia type |
| Study risk of bias assessment | 11 | Specify the methods used to assess risk of bias, including the tool(s) used and how many reviewers assessed each study. | 5-Methods — Cochrane risk-of-bias tool, six domains, two co-authors (DB, HA); disagreements resolved by DQT |
| Effect measures | 12 | Specify for each outcome the effect measure(s) used in the synthesis or presentation of results. | Not applicable — no quantitative meta-analysis; results synthesized narratively |
| Synthesis methods | 13a | Describe the processes used to decide which studies were eligible for each synthesis. | 4-5-Methods — studies grouped by comparator (intrathecal morphine, alternative blocks, PAI, PENG-as-adjunct) |
| Synthesis methods | 13b | Describe any methods required to prepare the data for presentation or synthesis. | Not applicable — narrative synthesis; no data transformation performed |
| Synthesis methods | 13c | Describe any methods used to tabulate or visually display results of individual studies and syntheses. | 5-6-Methods; Results — Tables 1–2, Figure 1 (risk of bias) |
| Synthesis methods | 13d | Describe any methods used to synthesize results and provide a rationale for the choice(s). | 5-Methods; Results — comparator-specific narrative synthesis (heterogeneous comparators precluded pooling) |
| Synthesis methods | 13e | Describe any methods used to explore possible causes of heterogeneity among study results. | 12-Discussion — heterogeneity discussed narratively (LA volume, PAI technique, timing of motor assessment) |
| Synthesis methods | 13f | Describe any sensitivity analyses conducted to assess robustness of the synthesized results. | Not applicable — no quantitative synthesis |
| Reporting bias assessment | 14 | Describe any methods used to assess risk of bias due to missing results in a synthesis. | Not applicable — no meta-analysis; publication/reporting bias addressed via inclusion criteria and discussed in Discussion |
| Certainty assessment | 15 | Describe any methods used to assess certainty (or confidence) in the body of evidence. | 12-Discussion — certainty appraised narratively; formal GRADE not applied |
| **RESULTS** | | | |
| Study selection | 16a | Describe the results of the search and selection process, ideally using a flow diagram. | 6-Results, para. 1; Figure 1 (PRISMA 2020 flow diagram): 468→114→44→10 |
| Study selection | 16b | Cite studies that met many but not all inclusion criteria and explain why they were excluded. | Results; Appendix A — 34 excluded full-text trials with reasons |
| Study characteristics | 17 | Cite each included study and present its characteristics. | Results; Tables 1–2 — characteristics of the 10 included trials [3–12] |
| Risk of bias in studies | 18 | Present assessments of risk of bias for each included study. | Results; Figure 2 — risk-of-bias summary |
| Results of individual studies | 19 | Present, for all outcomes, summary statistics for each group and the effect estimate for each study. | Not applicable |
| Results of syntheses | 20a | For each synthesis, briefly summarize the characteristics and risk of bias among contributing studies. | 6-12 Results (summary paragraphs); Discussion, para. 1 |
| Results of syntheses | 20b | Present results of all statistical syntheses; if meta-analysis was done, present summary estimate and precision. | Not applicable — no meta-analysis performed |
| Results of syntheses | 20c | Present results of all investigations of possible causes of heterogeneity. | 12-13-Discussion — sources of heterogeneity discussed narratively |
| Results of syntheses | 20d | Present results of all sensitivity analyses. | Not applicable |
| Reporting biases | 21 | Present assessments of risk of bias due to missing results for each synthesis. | 14-Discussion — addressed via registration/protocol-integrity inclusion criteria |
| Certainty of evidence | 22 | Present assessments of certainty in the body of evidence for each outcome. | 14-16-Discussion; Conclusion — evidence described as limited/heterogeneous |
| **DISCUSSION** | | | |
| Discussion | 23a | Provide a general interpretation of the results in the context of other evidence. | 14-15-Discussion — incl. new paragraph comparing with prior systematic reviews/meta-analyses |
| Discussion | 23b | Discuss any limitations of the evidence included in the review. | 16-Discussion — heterogeneity of comparators, small number of retained trials |
| Discussion | 23c | Discuss any limitations of the review processes used. | 16-Discussion — restrictive inclusion criteria; post-study registration limitation |
| Discussion | 23d | Discuss implications of the results for practice, policy, and future research. | 16-Discussion (inpatient vs outpatient THA); Conclusion; Table 3 (future directions) |
| **OTHER INFORMATION** | | | |
| Registration and protocol | 24a | Provide registration information, including register name and registration number, or state not registered. | 4-Methods; Abstract — INPLASY registration number: INPLASY202670024 (post-study registration) |
| Registration and protocol | 24b | Indicate where the review protocol can be accessed, or state that a protocol was not prepared. | 4-Methods — INPLASY record (https://inplasy.com/); DOI: 10.37766/inplasy2026.7.0024 |
| Registration and protocol | 24c | Describe and explain any amendments to information provided at registration or in the protocol. | 4-Methods/Discussion — review reframed from narrative to systematic during peer review; registered post-study |
| Support | 25 | Describe sources of financial or non-financial support, and the role of funders. | Funding statement — no specific grant received |
| Competing interests | 26 | Declare any competing interests of review authors. | Declaration of Conflicting Interests — none |
| Availability of data | 27 | Report which of the following are publicly available and where: data, analytic code, other materials. | Data Availability Statement — available upon reasonable request |
